# Fibrinogen-to-albumin ratio independently predicts left ventricular aneurysm in STEMI patients: a retrospective cohort study

**DOI:** 10.3389/fcvm.2026.1837371

**Published:** 2026-07-08

**Authors:** Qinshuo Zhao, Mengying He, Dong Hu, Wei Song, Xiao Li

**Affiliations:** 1 Department of Cardiology, Renmin Hospital of Wuhan University, Wuhan, China; 2 Department of Cardiology, The Central Hospital of Wuhan, Tongji Medical College, Huazhong University of Science and Technology, Wuhan, China; 3 Department of Pharmacy, Renmin Hospital of Wuhan University, Wuhan, China; 4 Department of Orthopedics, Renmin Hospital of Wuhan University, Wuhan, China

**Keywords:** acute ST-elevation myocardial infarction, fibrinogen-to-albumin ratio, inflammatory biomarker, left ventricular aneurysm, risk prediction

## Abstract

**Background:**

Left ventricular aneurysm (LVA) is a severe complication following acute ST-elevation myocardial infarction (STEMI) that worsens prognosis. The fibrinogen-to-albumin ratio (FAR) is an inflammatory biomarker with prognostic value in cardiovascular disease, but its role in predicting LVA remains unclear.

**Methods:**

We conducted a retrospective cohort study including 1,245 STEMI patients undergoing primary percutaneous coronary intervention (pPCI) at Renmin Hospital of Wuhan University between January 2020 and December 2025. An additional temporally independent cohort of 310 STEMI patients admitted between January 1, 2026 and June 15, 2026 was used for temporal validation. Restricted cubic spline analysis (RCS) was performed to examine the relationship between continuous FAR and LVA risk. Least absolute shrinkage and selection operator (LASSO) regression and multivariable logistic regression were used for variable selection and association analysis. Receiver operating characteristic (ROC) curve, calibration curve, decision curve analysis (DCA), and nomogram were used to develop and validate the prediction model.

**Results:**

In the development cohort, high FAR was independently associated with LVA (OR = 2.10, 95% CI: 1.09–4.04, *P* = 0.027). The combined model incorporating FAR, left ventricular ejection fraction (LVEF), and N-terminal pro-B-type natriuretic peptide (NT-proBNP) demonstrated high discrimination, with an area under the curve (AUC) of 0.933 (95% CI: 0.914–0.952). In the temporal validation cohort, the combined model retained excellent discriminative performance, with an AUC of 0.934 (95% CI: 0.894–0.974), and showed acceptable calibration, with a mean absolute error of 0.021 after 1,000 bootstrap resamples.

**Conclusion:**

Elevated FAR is independently associated with an increased risk of LVA in patients with STEMI following pPCI. Temporal validation further supported the stability of the prediction model in a later patient cohort.

## Introduction

Left ventricular aneurysm (LVA) is a severe complication after ST-elevation myocardial infarction (STEMI), occurring in 3%–15% of patients despite timely reperfusion (1, 2). The pathophysiology of LVA involves transmural myocardial necrosis, subsequent inflammatory response, and progressive ventricular remodeling, ultimately leading to aneurysmal dilation and thinning of the infarcted wall (3). Patients who develop LVA incur a substantially heightened risk of heart failure (4), malignant arrhythmias (5), thromboembolic events (6), and sudden cardiac death, with five-year mortality rates approaching 50% (7). Early identification of high-risk patients is therefore critical for improving outcomes.

Inflammation and coagulation activation are key drivers of ventricular remodeling and LVA formation (8–11). Persistent and excessive inflammatory responses disrupt extracellular matrix metabolism, weaken myocardial tensile strength, and promote regional wall thinning, ultimately leading to LVA formation (3, 12). Therefore, inflammatory-coagulative biomarkers that reflect the intensity of systemic inflammation may serve as potential predictors for LVA.

The FAR is a composite inflammatory biomarker that integrates pro-inflammatory (fibrinogen) and anti-inflammatory (albumin) components, which has shown prognostic value in cardiovascular diseases (13–18). Recent studies have shown that elevated FAR is associated with poor prognosis in various cardiovascular conditions, including acute coronary syndrome (19), heart failure (20), and atrial fibrillation (21). Although FAR has shown predictive value for major adverse cardiovascular events and all-cause mortality in STEMI patients undergoing pPCI (22), its predictive capacity for LVA in these patient population remains unexplored.

This study aimed to develop and temporally validate a clinical prediction model for LVA in STEMI patients undergoing pPCI.

## Methods

### Study design

This study was conducted as a retrospective clinical prediction model development and temporal validation study. The development cohort included 1,245 consecutive patients with STEMI who underwent pPCI between January 2020 and December 2025. An independent temporal validation cohort consisting of 310 consecutive STEMI patients admitted between January 1, 2026 and June 15, 2026 was subsequently established. The temporal validation cohort was not involved in variable selection, model fitting, or nomogram construction, and was used only to evaluate the temporal generalizability of the established prediction model. STEMI was diagnosed according to the Fourth Universal Definition of Myocardial Infarction (23). The study protocol was approved by the Clinical Research Ethics Committee of Renmin Hospital of Wuhan University (WDRY2026-K054), and informed consent was waived due to the retrospective design.

### Study population

**Figure 1:**
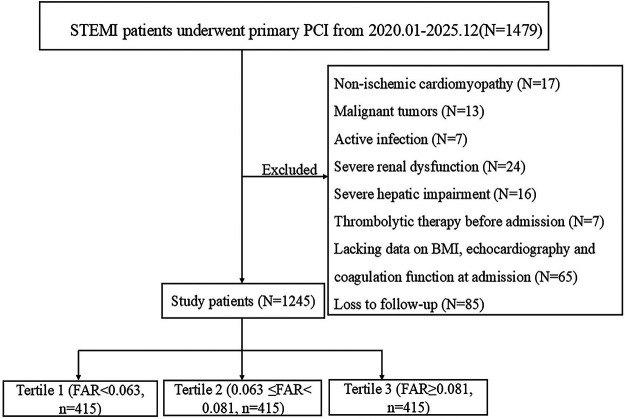
Flow diagram of patient selection in the development cohort. STEMI, ST-segment elevation myocardial infarction; PCI, percutaneous coronary intervention; FAR, fibrinogen-to-albumin ratio.

A total of 1,479 consecutive patients diagnosed with STEMI who underwent pPCI were screened between January 2020 and December 2025 in the development cohort. After applying the exclusion criteria, 1,245 patients were ultimately enrolled in this retrospective cohort study. The detailed patient selection flowchart is presented in Figure 1. The same inclusion and exclusion criteria were applied to the temporal validation cohort, which consisted of 310 consecutive STEMI patients admitted between January 1, 2026 and June 15, 2026.

### Inclusion and exclusion criteria

Inclusion criteria were: (1) age ≥18 years; (2) diagnosis of STEMI according to the universal definition; (3) emergency PCI performed within 24 h of symptom onset; (4) complete laboratory, echocardiographic, and follow-up data available. Exclusion criteria were: (1) non-ischemic cardiomyopathy (such as hypertrophic, takotsubo, and dilated cardiomyopathy); (2) congenital heart disease or severe valvular heart disease; (3) active infection, autoimmune disease, malignant tumor, or severe hepatic/renal dysfunction; (4) incomplete echocardiographic or laboratory data; (5) received thrombolytic therapy before admission or lack of PCI treatment.

### Data collection

This was a retrospective analysis using data that had been prospectively recorded in the hospital’s electronic medical record (EMR) system.

Data extraction was performed independently by two researchers to minimize bias. Any discrepancies were resolved by a senior cardiologist. To ensure data integrity, extracted variables were cross-verified against original source documents, including laboratory reports, echocardiograms, and procedural notes.

### Variables and definitions

1. Demographics: Data collected at admission included age, sex, body mass index (BMI, calculated as weight/height^2^), and heart rate.
2. Clinical History: This encompassed hypertension (defined as systolic blood pressure (SBP) ≥ 140 mmHg, diastolic ≥90 mmHg, or use of antihypertensive agents), type 2 diabetes mellitus (defined as fasting plasma glucose (FPG) ≥ 7.0 mmol/L, HbA1c (%) ≥ 6.5%, or use of antidiabetic medication), current smoking, alcohol use, Killip classification, and discharge medications.
3. Laboratory Parameters: Blood samples were obtained within 2 h after admission and analyzed in the central laboratory using standardized assays. Key measures included: (1) Fibrinogen (FIB) and albumin (ALB), which were quantified using the Clauss method and bromocresol green method, respectively. The FAR index was calculated as FIB (g/L)/ALB (g/L). Patients were subsequently categorized into tertiles based on the FAR distribution. (2) Cardiac Biomarkers: NT-proBNP and high-sensitivity cardiac troponin I (hs-cTnI) were measured by chemiluminescent immunoassay.
4. Other Data: Additional laboratory data included complete blood count, renal and liver function tests, lipid profile, and glycated hemoglobin (HbA1c).

### Echocardiographic assessments

Two-dimensional transthoracic echocardiography (TTE) was performed on all patients within three days of admission and repeated at 1 and 3 months of follow-up. All examinations were conducted by two experienced echocardiographers (each with >5 years of clinical experience) who were blinded to patients’ FAR levels and clinical outcomes.

The diagnosis of LVA was established based on the Coronary Artery Surgery Study (CASS) criteria, consistent with contemporary definitions for mechanical complications of acute myocardial infarction (24). Standard echocardiographic parameters were recorded, including left ventricular end-diastolic diameter (LVEDD), left atrial diameter (LAD), interventricular septal thickness (IVSD), and the presence of regional wall motion abnormalities.

Cardiac MRI and ventriculography were not routinely performed in all patients and therefore were not used as systematic diagnostic confirmation methods for LVA in this retrospective cohort.

### Coronary angiography and clinical management

All patients received dual antiplatelet therapy (aspirin 300 mg loading dose, then 100 mg daily; clopidogrel 600 mg loading dose, then 75 mg daily; or ticagrelor 180 mg loading dose, then 90 mg twice daily) and intravenous unfractionated heparin (100 U/kg) prior to pPCI. Two independent interventional cardiologists reviewed coronary angiograms to identify culprit vessels based on angiographic findings and electrocardiographic localization. Coronary artery disease severity was quantified using the Gensini score (25). Symptom-to-balloon (S2B) time, stent type, and stent number were recorded. Detailed procedural protocols have been reported previously (25).

### Nomogram construction and validation

A clinical nomogram was developed to predict the probability of LVA formation in patients with STEMI, based on the results of multivariable logistic regression. Only variables identified as independent predictors were incorporated into the nomogram, including Killip grade ≥ II, IRA-LAD, FAR, NT-proBNP, LVEF, and LVEDD. Variables included in the nomogram were consistent with those retained by LASSO regression and confirmed as independent predictors in multivariable logistic regression.

The performance of the nomogram was evaluated using several statistical approaches. Discrimination was assessed using the AUC. Calibration was evaluated using the calibration curve with 1000 bootstrap resamples to correct for overfitting.

Furthermore, DCA was conducted to assess the clinical utility of the nomogram by quantifying the net benefit at different threshold risk probabilities, compared with the “treat-all” and “treat-none” strategies.

### Statistical analysis

Prior to statistical analysis, standard data cleaning procedures were performed. Missing values in the dataset were handled according to their missing rates and types. Continuous variables with a low missing rate (< 5%) were imputed with the median, and categorical variables were imputed with the mode. For variables with a moderate missing rate (5%–20%), multiple imputation was employed to maintain data integrity. Variables with a high missing rate (> 20%) were excluded from the final analysis to ensure the robustness of the results. Outliers were detected using boxplots and Z-scores (|Z| > 3.0) and verified against original medical records. All data cleaning and quality control procedures were performed using R software 4.4.1 (R Foundation for Statistical Computing, Vienna, Austria).

Statistical analyses were performed using SPSS 26.0 (IBM Corp., Armonk, NY, USA) and R 4.4.1. A two-sided *P* < 0.05 was considered statistically significant. Continuous variables were expressed as mean ± SD or median (Q1–Q3) and compared using Student's t-test or Mann–Whitney U-test, respectively. Categorical variables were presented as numbers (percentages) and analyzed using chi-square or Fisher's exact test.

To avoid overfitting and ensure model stability, LASSO regression with 10-fold cross-validation was used for variable selection prior to multivariable modeling. All candidate variables were initially prespecified based on clinical relevance and prior literature, followed by LASSO for data-driven selection. The regularization parameter *λ* was determined using the 1-standard error criterion, and all continuous variables were standardized before analysis. Variables retained in the LASSO model were included in subsequent multivariable logistic regression to identify independent predictors of LVA. Results are reported as odds ratios (ORs) with 95% confidence intervals (CIs). Collinearity was assessed using the variance inflation factor (VIF), with values < 5 indicating no significant collinearity.

Guideline-directed medical therapies, including statins, angiotensin-converting enzyme inhibitors/angiotensin receptor–neprilysin inhibitors (ACEI/ARNI), beta-blockers, and mineralocorticoid receptor antagonists (MRA), were considered as potential confounding variables. These variables were included in the list of candidate covariates for univariable analysis and subsequent multivariable logistic regression to adjust for their potential influence on left ventricular remodeling and LVA formation.

The predictive performance of the final model was evaluated using ROC curve analysis, with the optimal cutoff determined by the Youden index. Model discrimination was quantified using the AUC. Calibration was assessed using calibration curves with 1000 bootstrap resamples, and internal validation was performed using bootstrap validation to minimize overfitting. DCA was conducted to evaluate the net clinical benefit of the prediction model across different threshold probabilities. RCS analysis was used to explore the potential nonlinear relationship between the FAR and LVA risk, adjusted for age, gender, LVEF, and NT-proBNP. Both RCS analysis and tertile-based subgroup analysis were pre-specified in the statistical analysis plan before data analysis.

A clinical nomogram was constructed based on the independent predictors identified in the multivariable model. All statistical procedures adhered to standard methodological principles for predictive modeling and observational research.

### Temporal validation

For temporal validation, the final prediction model derived from the development cohort was applied unchanged to the temporal validation cohort. No variable reselection or model refitting was performed in the validation cohort. Discrimination was assessed using ROC curve analysis and quantified by the AUC with 95% CI. Calibration was evaluated using calibration curves and mean absolute error after 1,000 bootstrap resamples. DCA was performed to assess the clinical net benefit of the model in the temporal validation cohort. Baseline characteristics between the development and temporal validation cohorts were compared and are presented in Supplementary Table 1.

The predictive performance of individual predictors and the combined model in the temporal validation cohort was summarized in Table 5. In addition, RCS analysis was performed in the temporal validation cohort as an exploratory analysis to examine whether the direction of the association between FAR and LVA risk was consistent with that observed in the development cohort.

## Results

### RCS analysis to explore the relationship between FAR and LVA risk

**Figure 2:**
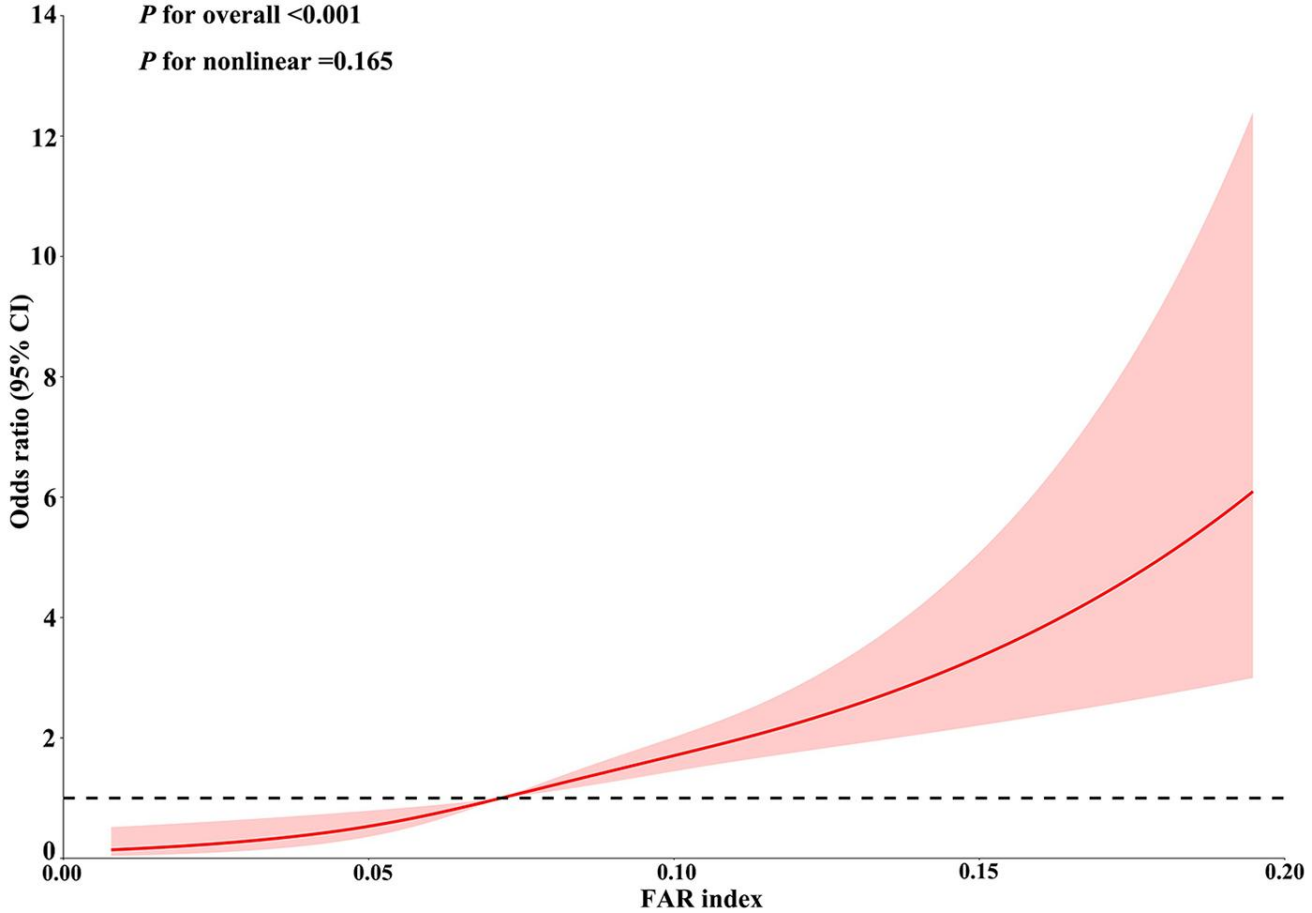
RCS model showing the links between the FAR index and the risk of LVA. RCS, restricted cubic spline; FAR, fibrinogen-to-albumin ratio; LVA, left ventricular aneurysm; OR, odds ratio; CI, confidence interval.

As pre-specified in the statistical analysis plan, RCS analysis was performed in the development cohort to examine the association between continuous FAR values and the risk of LVA. As shown in Figure 2, the risk of LVA increased progressively with higher FAR levels, with a significant overall association (*P* for overall < 0.001). No significant evidence of nonlinearity was observed (*P* for nonlinearity = 0.165), suggesting an approximately linear dose-response relationship between FAR and LVA risk in the development cohort.

### LASSO regression for variable selection

**Figure 3:**
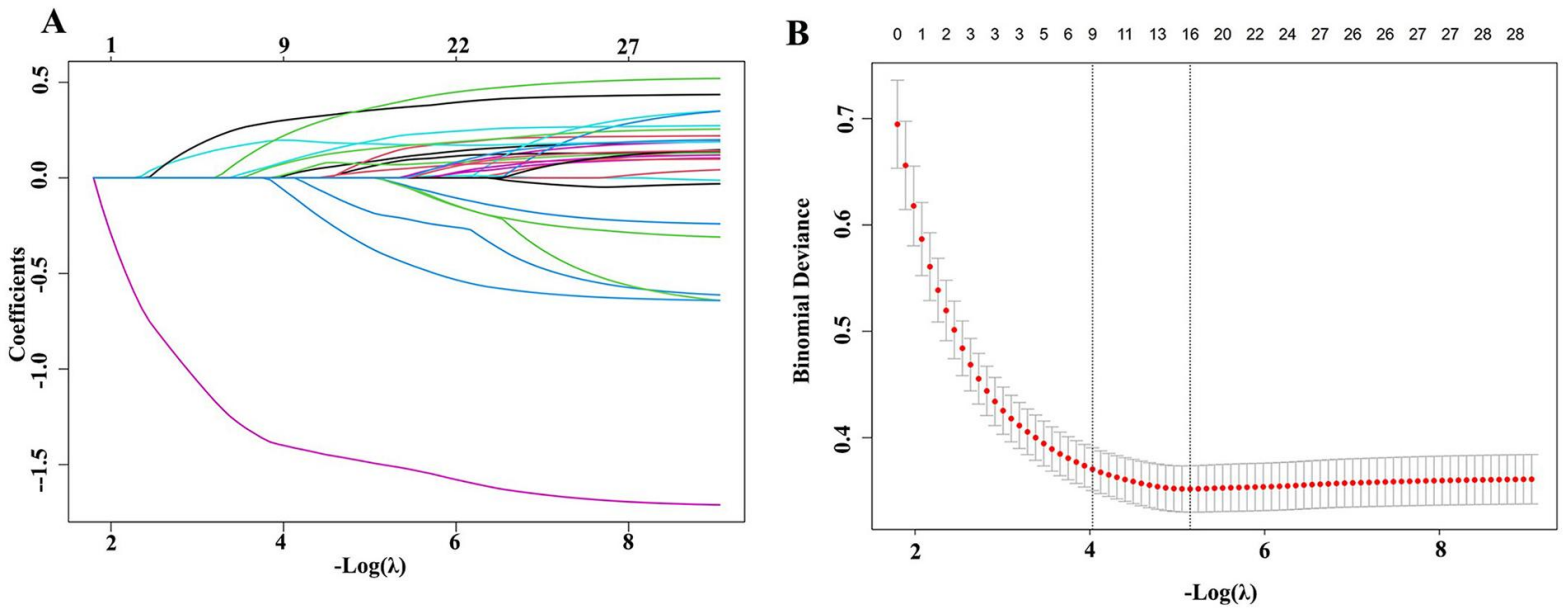
Variable selection using LASSO regression. **(A)** Coefficient profiles of all input variables. Each colored line represents the changing coefficient of one predictor as *λ* increases. **(B)** Binomial deviance curve from 10-fold cross-validation. The left dashed line indicates the *λ* with minimum binomial deviance, and the right dashed line corresponds to the optimal *λ* determined by the 1-standard error rule.

To screen candidate risk factors for LVA, we performed LASSO regression with 10-fold cross-validation. Figure 3A illustrates the coefficient shrinkage trajectories of all included variables as the penalty parameter *λ* increased. With rising -log(*λ*), the regression coefficients of non-essential predictors gradually compressed toward zero, while key variables retained stable non-zero coefficients.

Figure 3B displayed the binomial deviance curve across different *λ* values. The two vertical dashed lines represent the optimal *λ* selected by the minimum deviance criterion and the 1-standard error (1-SE) criterion, respectively. Ultimately, a total of 9 variables with non-zero coefficients were retained after LASSO penalization. These screened independent predictors were subsequently entered into the multivariable regression model. Statistically significant predictors identified from this model were finally used for nomogram construction to ensure clinical practicability. This sequential approach effectively reduced model complexity, mitigated overfitting, and improved the stability and generalizability of our prediction framework.

### Baseline characteristics

As a pre-specified complementary analysis, patients were divided into three groups based on FAR tertiles. The cutoff values for FAR tertiles were T1 < 0.063, T2 0.063–0.081, T3 > 0.081. Baseline characteristics are shown in Table 1. The overall cohort had a mean age of 59.69 ± 12.12 years, with 83.5% male. Patients in the highest FAR tertile (T3) were significantly older (63.22 ± 11.37 vs. 56.13 ± 12.12 years, *P* < 0.001) and had a lower male proportion (77.8% vs. 88.7%, *P* < 0.001) compared with the lowest tertile (T1).

**Table 1:** Baseline characteristics of the study population stratified by tertiles.

Variables	Total (*n* = 1245)	Tertile 1 (*n* = 415)	Tertile 2 (*n* = 415)	Tertile 3 (*n* = 415)	*P*-value
Demographics					
Age (years)	59.69 ± 12.12	56.13 ± 12.12	59.72 ± 11.82	63.22 ± 11.37	<0.001
Gender (male, %)	1040 (83.5%)	368 (88.7%)	349 (84.1%)	323 (77.8%)	<0.001
Height (cm)	169.00 (164.00, 172.00)	170.00 (165.00, 173.00)	169.00 (164.00, 172.00)	168.00 (162.00, 171.00)	<0.001
Weight (kg)	70.00 (62.00,78.00)	71.00 (65.00, 80.00)	70.00 (62.00, 77.00)	66.00 (60.00, 75.00)	<0.001
BMI (kg/m^2^)	24.62 (22.84, 26.73)	25.00 (23.51, 27.02)	24.74 (22.80, 26.78)	24.22 (22.04, 26.57)	<0.001
HR (times/min)	81.76 ± 14.98	81.64 ± 14.73	80.70 ± 14.30	82.94 ± 15.82	0.096
SBP (mmHg)	122.35 ± 18.92	122.52 ± 17.86	122.29 ± 19.28	122.22 ± 19.61	0.973
DBP (mmHg)	76.81 ± 12.76	77.36 ± 11.85	76.55 ± 13.07	76.53 ± 13.31	0.559
Current smoker, *n* (%)	883 (70.9%)	302 (72.8%)	293 (70.6%)	288 (69.4%)	0.555
Current drinker, *n* (%)	706 (56.7%)	231 (55.7%)	243 (58.6%)	232 (55.9%)	0.647
Laboratory data					
WBC ( × 10^9^/L)	10.91 (9.01, 13.03)	10.65 (9.02, 13.00)	10.97 (8.93, 12.88)	11.17 (9.12, 13.33)	0.250
Neutrophil (10^9^/L)	8.82 (7.22,11.06)	8.70 (7.27, 11.09)	8.73 (7.08, 11.00)	9.05 (7.31, 11.12)	0.426
Lymphocyte ( × 10^9^/L)	1.12 (0.82,1.56)	1.13 (0.85,1.51)	1.14 (0.82,1.58)	1.12 (0.79,1.57)	0.518
Monocyte ( × 10^9^/L)	0.55 (0.39,0.77)	0.50 (0.36,0.70)	0.54 (0.39,0.74)	0.64 (0.42,0.91)	<0.001
Platelet count (10^9^/L)	220.00 (181.00,264.00)	223.00 (186.00,262.50)	217.00 (178.00,265.00)	220.00 (179.50,265.00)	0.735
RBC ( × 10^9^/L)	4.58 ± 0.59	4.68 ± 0.53	4.61 ± 0.58	4.46 ± 0.64	<0.001
Hemoglobin (g/L)	140.45 ± 18.02	144.23 ± 16.58	141.14 ± 17.37	135.97 ± 19.08	<0.001
Albumin (g/L)	40.10 (38.00,42.20)	41.07 (39.00,43.30)	40.30 (38.50,41.90)	38.76 (36.65,40.86)	<0.001
GLB(g/L)	24.90 (22.68,27.50)	24.70 (22.34,27.34)	24.40 (22.48,27.16)	25.50 (23.24,28.35)	<0.001
ALT (U/L）	39.00 (25.00,59.00)	40.00 (25.24,60.91)	37.00 (25.00,57.00)	39.00 (24.00,59.00)	0.371
AST (U/L）	130.12 (57.00,247.00)	128.00 (56.00,247.50)	123.64 (58.50,239.90)	137.00 (58.50,258.45)	0.447
UA (umol/L)	371.00 (310.00,443.00)	369.02 (306.15,446.00)	363.00 (303.00,422.00)	379.89 (317.50,457.04)	0.003
Cr (umol/L)	70.00 (60.00,82.10)	68.00 (59.00,79.00)	68.00 (59.00,80.57)	73.16 (62.00,93.00)	<0.001
BUN (mmol/L)	5.76 (4.70,6.99)	5.65 (4.64,6.42)	5.56 (4.59,6.70)	6.30 (5.01,7.80)	<0.001
eGFR (ml/min/1.73 m^2^)	92.57 ± 20.47	98.20 ± 17.95	94.68 ± 17.35	84.83 ± 23.23	<0.001
FBG (mmol/L)	7.13 (6.03,9.18)	7.00 (6.07,8.63)	7.02 (6.06,8.82)	7.31 (5.98,10.11)	0.079
TG (mmol/L)	0.90 (0.61,1.53)	0.90 (0.62,1.63)	0.86 (0.57,1.47)	0.94 (0.63,1.48)	0.327
TC (mmol/L)	4.50 (3.88,5.17)	4.50 (3.83,5.16)	4.51 (3.92,5.09)	4.52 (3.88,5.28)	0.58
LDL-C (mmol/L)	2.86 (2.29,3.47)	2.85 (2.29,3.47)	2.88 (2.31,3.45)	2.89 (2.26,3.50)	0.855
HDL-C (mmol/L)	0.98 (0.83,1.16)	0.98 (0.84,1.15)	0.98 (0.82,1.16)	0.97 (0.82,1.17)	0.789
sdLDL	0.99 (0.68,1.25)	0.99 (0.74,1.31)	1.00 (0.68,1.27)	0.96 (0.64,1.20)	0.020
Lp(a)	137.00 (69.47,294.00)	116.00 (63.50,253.65)	128.00 (65.00,269.50)	174.00 (89.00,351.12)	<0.001
CK (U/L)	1556.00 (661.00,3180.00)	1643.00 (628.80,3112.14)	1504.00 (710.00,3112.00)	1486.00 (657.00,3243.00)	0.918
LDH (U/L)	419.00 (270.00,655.00)	384.00 (251.00,603.48)	391.00 (264.50,630.50)	484.00 (301.00,738.24)	<0.001
Fibrinogen (g/L)	2.88 (2.42,3.44)	2.23 (1.94,2.43)	2.88 (2.70,3.08)	3.81 (3.42,4.54)	<0.001
D-dimer (ng/mL)	0.37 (0.21,0.70)	0.30 (0.18,0.54)	0.34 (0.19,0.62)	0.53 (0.30,1.02)	<0.001
cTnI > 50 ng/mL	725 (58.2%)	235 (56.6%)	242 (58.3%)	248 (59.8%)	0.657
NT-proBNP (pg/mL)	267.00 (95.00,1023.00)	136.00 (59.00,486.50)	212.00 (90.50,757.00)	720.00 (202.00,2436.00)	<0.001
HbAlc (%)	6.10 (5.70,6.80)	6.00 (5.70,6.50)	6.10 (5.80,6.80)	6.10 (5.80,7.10)	<0.001
Sodium (mmol/L)	139.10 ± 2.96	139.24 ± 2.87	139.03 ± 2.86	139.03 ± 3.14	0.508
Potassium (mmol/L)	4.03 ± 0.41	3.97 ± 0.36	4.00 ± 0.38	4.12 ± 0.46	<0.001
Echocardiography					
AAOD (mm)	33.68 ± 3.50	33.45 ± 3.63	33.60 ± 3.51	34.00 ± 3.35	0.062
LAD (mm)	35.71 ± 4.09	35.58 ± 4.15	35.45 ± 3.81	36.08 ± 4.27	0.062
LVEDD (mm)	47.08 ± 4.40	46.90 ± 4.09	46.81 ± 4.48	47.52 ± 4.61	0.041
RAD (mm)	33.68 ± 4.04	33.78 ± 4.03	33.60 ± 4.16	33.67 ± 3.95	0.825
IVSD (mm)	10.19 ± 1.23	10.20 ± 1.23	10.11 ± 1.20	10.27 ± 1.27	0.151
LVEF (%)	50.71 ± 7.55	51.81 ± 6.92	51.33 ± 7.53	48.99 ± 7.89	<0.001
Medical history					
HBP, *n* (%)	665 (53.4%)	201 (48.4%)	215 (51.8%)	249 (60.0%)	0.003
DM, *n* (%)	275 (22.1%)	66 (15.9%)	100 (24.1%)	109 (26.3%)	<0.001
Hyperlipidemia, *n* (%)	1149 (92.3%)	381 (91.8%)	384 (92.5%)	384 (92.5%)	0.903
Renal insufficiency	1213 (97.4%)	409 (98.6%)	405 (97.6%)	399 (96.1%)	0.087
CHD, *n* (%)	133 (10.7%)	34 (8.2%)	51 (12.3%)	48 (11.6%)	0.125
Stroke, *n* (%)	71 (5.7%)	18 (4.3%)	26 (6.3%)	27 (6.5%)	0.336
Drink, *n* (%)	706 (56.7%)	231 (55.7%)	243 (58.6%)	232 (55.9%)	0.555
Smoking, *n* (%)	706 (56.7%)	231 (55.7%)	243 (58.6%)	232 (55.9%)	0.647
Medication at Hospital discharge					
Aspirin, *n* (%)	1194 (95.9%)	407 (98.1%)	396 (95.4%)	391 (94.2%)	0.016
Clopidogrel, *n* (%)	317 (25.5%)	81 (19.5%)	105 (25.3%)	131 (31.6%)	<0.001
Ticagrelor, *n* (%)	920 (73.9%)	331 (79.8%)	308 (74.2%)	281 (67.7%)	<0.001
Statins, *n* (%)	1239 (99.52%)	412 (99.28%)	414 (99.76%)	413 (99.52%)	0.914
β-blockers, *n* (%)	1039 (83.5%)	353 (85.1%)	340 (81.9%)	346 (83.4%)	0.478
Thiazide/loop diuretic, *n* (%)	402 (32.3%)	91 (21.9%)	129 (31.1%)	182 (43.9%)	<0.001
Spironolactone, *n* (%)	425 (34.1%)	101 (24.3%)	136 (32.8%)	188 (45.3%)	<0.001
ACEI, *n* (%)	122 (9.8%)	42 (10.1%)	42 (10.1%)	38 (9.2%)	0.865
ARB, *n* (%)	687 (55.2%)	224 (54.0%)	234 (56.4%)	229 (55.2%)	0.784
OADS, *n* (%)	335 (26.9%)	87 (21.0%)	115 (27.7%)	133 (32.0%)	0.001
Coronary artery injury, *n* (%)					
LM, *n* (%)	6 (0.5%)	1 (0.2%)	3 (0.7%)	2 (0.5%)	0.605
LAD, *n* (%)	642 (51.6%)	199 (48.0%)	217 (52.3%)	226 (54.5%)	0.161
LCX, *n* (%)	171 (13.7%)	51 (12.3%)	57 (13.7%)	63 (15.2%)	0.481
RCA, *n* (%)	380 (30.5%)	144 (34.7%)	126 (30.4%)	110 (26.5%)	0.037
Gensini score	48.00 (34.00,76.00)	46.00 (32.00,69.00)	48.00 (34.00,74.00)	53.00 (39.00,80.00)	<0.001
Clinical management					
S2B (h)	5.00 (3.00,10.00)	4.00 (2.00,8.00)	5.00 (3.00,9.00)	6.00 (3.50,16.00)	<0.001
Mechanical support use	201 (16.1%)	45 (10.8%)	55 (13.3%)	101 (24.3%)	<0.001
Killip class ≥2, *n* (%)	946 (76.0%)	346 (83.4%)	326 (78.6%)	274 (66.0%)	<0.001
Stent implantation	759 (61.0%)	262 (63.1%)	239 (57.6%)	258 (62.2%)	0.217

Presented as mean ± standard deviation (SD) for normally distributed data or median (interquartile range, IQR) for non-normally distributed data. Categorical variables were presented as *n* (%) (number of patients with the characteristic, percentage of the subgroup).

BMI, body mass index (kg/m^2^); HR, heart rate (times/min); SBP, systolic blood pressure (mmHg); DBP, diastolic blood pressure (mmHg); HBP, hypertension; DM, diabetes mellitus; CHD, coronary heart disease; Killip class, clinical classification of heart failure severity after myocardial infarction; UA, uric acid (umol/L); Cr, creatinine (umol/L); BUN, blood urea nitrogen (mmol/L); eGFR, estimated glomerular filtration rate (ml/min/1.73 m^2^); FBG, fasting blood glucose (mmol/L); HbA1c (%), glycated hemoglobin (%); Sodium, serum sodium (mmol/L); Potassium, serum potassium (mmol/L); ALT, alanine aminotransferase (U/L); AST, aspartate aminotransferase (U/L); TG, triglycerides (mmol/L); TC, total cholesterol (mmol/L); LDL-C, low-density lipoprotein cholesterol (mmol/L); HDL-C, high-density lipoprotein cholesterol (mmol/L); sdLDL, small dense low-density lipoprotein (mmol/L); Lp(a), lipoprotein(a) (mg/dL); CK, creatine kinase (U/L); LDH, lactate dehydrogenase (U/L); cTnI, cardiac troponin I (ng/ml); NT-proBNP, N-Terminal Pro-B-Type Natriuretic Peptide (pg/mL); WBC, white blood cell count ( × 10⁹/L); Neutrophil, neutrophil count ( × 10⁹/L); Lymphocyte, lymphocyte count ( × 10⁹/L); Monocyte, monocyte count ( × 10⁹/L); RBC, red blood cell count ( × 10⁹/L); AAOD, ascending aorta outer diameter (mm); LAD, left atrial diameter (mm); LVEDD, left ventricular end-diastolic diameter (mm); RAD, right atrial diameter (mm); IVSD, interventricular septal thickness (mm); LVEF, left ventricular ejection fraction (%); LM: left main coronary artery; LAD, left anterior descending artery; LCX, left circumflex artery; RCA, right coronary artery; Gensini score, quantitative scoring system for coronary artery stenosis severity; S2B (h): time from symptom onset to balloon dilation (hours); ACEI, angiotensin-converting enzyme inhibitor; ARB, angiotensin II receptor blocker; OADS, oral antidiabetic drugs.

Higher FAR was associated with significantly lower anthropometric parameters (weight, height, BMI; all *P* < 0.001). In contrast, admission SBP and DBP were comparable across tertiles (SBP: 122.52 ± 17.86 vs. 122.29 ± 19.28 vs. 122.22 ± 19.61 mmHg; DBP: 77.36 ± 11.85 vs. 76.55 ± 13.07 vs. 76.53 ± 13.31 mmHg; all *P* > 0.05). Admission heart rate (HR) also showed no significant difference across tertiles (*P* = 0.096).

Laboratory differences included higher monocyte counts, urea, creatinine, D-dimer, and NT-proBNP in T3 (all *P* < 0.001), but lower hemoglobin, albumin, and eGFR (all *P* < 0.001). FIB levels increased stepwise across tertiles (2.23 [1.94–2.43] vs. 2.88 [2.70–3.08] vs. 3.81 [3.42–4.54] g/L, *P* < 0.001). Conventional lipid parameters were comparable across tertiles, but small dense low-density lipoprotein cholesterol (sdLDL-C) was lower in T3 (*P* = 0.020) and Lp(a) increased progressively (*P* < 0.001).

Echocardiographic findings showed lower LVEF in T3 (48.99 ± 7.89% vs. 51.81 ± 6.92% and 51.33 ± 7.53% in T1 and T2, *P* < 0.001) and modestly larger LVEDD (*P* = 0.041).

Medical history showed increasing prevalence of HBP (48.4% vs. 51.8% vs. 60.0%, *P* = 0.003) and diabetes (15.9% vs. 24.1% vs. 26.3%, *P* < 0.001) across tertiles, but decreasing proportion of Killip class ≥ II (83.4% vs. 78.6% vs. 66.0%, *P* < 0.001).

Discharge medications differed by tertile: T3 patients were more frequently prescribed clopidogrel (31.6% vs. 19.5% in T1, *P* < 0.001), thiazide/loop diuretics, and spironolactone (both *P* < 0.001), but less frequently prescribed ticagrelor (67.7% vs. 79.8% in T1, *P* < 0.001).

Coronary angiographic characteristics showed less frequent RCA culprit vessels in T3 (26.5% vs. 34.7% in T1, *P* = 0.037), higher Gensini scores (53.00 [39.00–80.00] vs. 46.00 [32.00–69.00] in T1, *P* < 0.001), prolonged S2B time (*P* < 0.001), and more frequent mechanical support device use (24.3% vs. 10.8% in T1, *P* < 0.001).

### FAR index and the incidence of LVA

**Figure 4:**
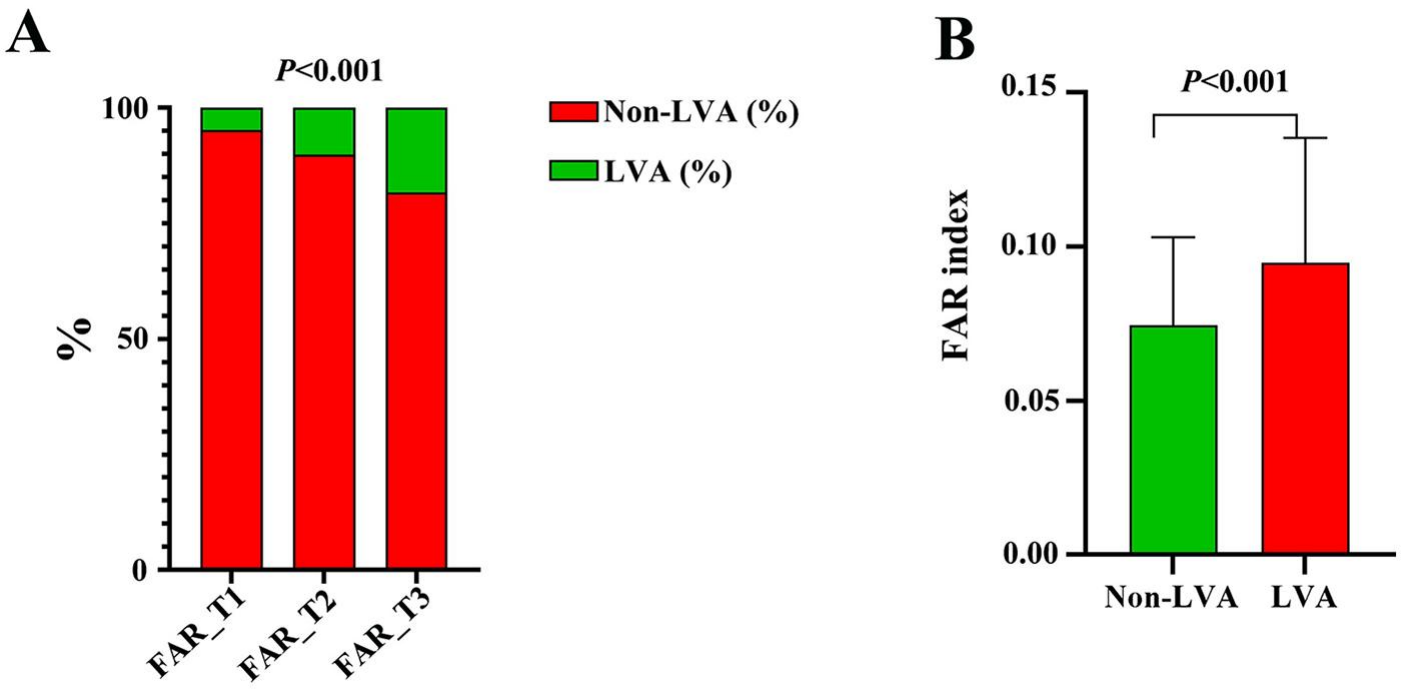
LVA incidence stratified by FAR tertiles and the distribution of FAR across groups. **(A)** Bar chart depicting the incidence of LVA across FAR tertiles. **(B)** Box plot showing the distribution of FAR levels in patients with and without LVA.

The incidence of LVA increased significantly with ascending FAR tertiles (3.6% vs. 10.8% vs. 18.8%; *P* < 0.001), supporting a positive dose-response relationship (Figures 4A,B).

### Subgroup analysis and interaction tests

**Figure 5:**
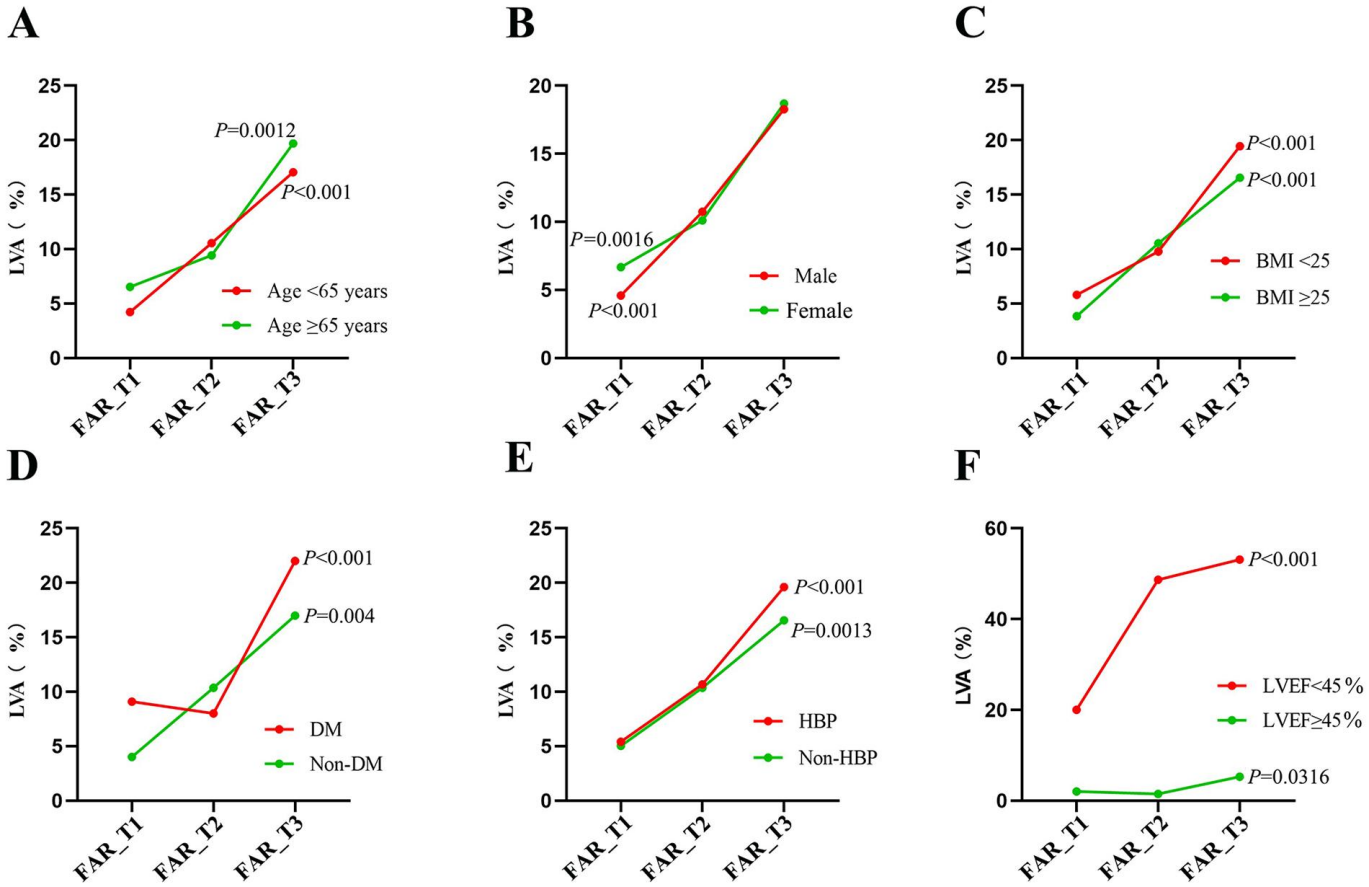
Incidence of LVA stratified by FAR tertiles across different clinical subgroups. **(A)** Age subgroups: <65 years (*n* = 781) and ≥65 years (*n* = 464). LVA incidence increased progressively with ascending FAR tertiles in both subgroups (*P* < 0.001 for <65 years; *P* = 0.001 for ≥65 years). **(B)** Gender subgroups: Male (*n* = 1040) and Female (*n* = 205). A significant positive trend between FAR tertiles and LVA incidence was observed in males (*P* < 0.0001) and females (*P* = 0.0016), with no significant interaction between gender and FAR (*P* for interaction = 0.18). **(C)** BMI subgroups: <25 kg/m^2^ (*n* = 684) and ≥25 kg/m^2^ (*n* = 561). LVA incidence rose significantly with higher FAR tertiles in both BMI categories (both *P* < 0.001). **(D)** DM subgroups: With DM (*n* = 275) and without DM (*n* = 970). A marked increase in LVA incidence across FAR tertiles was noted in both subgroups (both *P* < 0.001). **(E)** HBP subgroups: With HBP (*n* = 665) and without HBP (*n* = 580). LVA incidence increased significantly with ascending FAR tertiles in both subgroups (both *P* < 0.001). **(F)** LVEF subgroups: <45% (*n* = 254) and ≥45% (*n* = 991). The association between FAR and LVA incidence was most pronounced in the LVEF < 45% subgroup (*P* < 0.001), with a significant positive trend also observed in the LVEF ≥ 45% subgroup (*P* = 0.0316). FAR, fibrinogen-to-albumin ratio; LVA, left ventricular aneurysm; BMI, body mass index; DM, diabetes mellitus; HBP, hypertension; LVEF, left ventricular ejection fraction.

Subgroup analyses consistently demonstrated the robustness of this association across key patient characteristics (Figure 5). The positive trend remained statistically significant (all *P* for trend < 0.001) in strata defined by age (<65 and ≥65 years), gender (male and female), BMI (<25 and ≥25 kg/m^2^), diabetes mellitus status, and hypertension status. Notably, the absolute LVA incidence was higher in females and in patients with diabetes across all FAR tertiles. However, tests for interaction were non-significant for gender (*P* = 0.18), suggesting no significant effect modification by gender.

The association was particularly strong in patients with reduced LVEF (LVEF ≤45%). In this high-risk subgroup, LVA incidence rose markedly from 20.0% in FAR_T1 to 48.7% in FAR_T2 and 53.1% in FAR_T3 (*P* < 0.001). A significant, though less pronounced, positive trend was also maintained in patients with LVEF >45% (*P* < 0.001).

Because multiple subgroup analyses were conducted, these findings should be interpreted as exploratory and hypothesis-generating. We therefore focused primarily on the overall consistency of the association and interaction tests rather than isolated subgroup-specific *P* values.

### Correlation between FAR index and predictive indicators of LVA

Spearman's correlation analysis examined associations between FAR and potential LVA predictors (Table 2). FAR correlated positively with age ≥65 years (r = 0.205), hypertension (r = 0.112), and diabetes (r = 0.135), and negatively with male sex (r = −0.131) and BMI ≥25 kg/m^2^ (r = −0.082) (all *P* < 0.01).

**Table 2:** Spearman correlation analysis of the FAR index with clinical, laboratory, and echocardiographic variables.

Variables	CI (r)	*P*-value
Age ≥65 years	0.205	<0.001
HBP	0.112	<0.001
Gender (male = 1, female = 0)	−0.131	<0.001
DM	0.135	<0.001
BMI ≥25 kg/m^2^	−0.082	0.004
LVEDD	0.163	<0.001
Albumin (g/L)	−0.639	<0.001
Fibrinogen (g/L)	0.875	<0.001
D-dimer (ng/mL)	0.389	<0.001
Monocyte	0.237	<0.001
Gensini score	0.268	<0.001

FAR, fibrinogen-to-albumin ratio; STEMI, ST-segment elevation myocardial infarction; NT-proBNP, N-terminal pro-B-type natriuretic peptide; HBP, hypertension; DM, diabetes mellitus; LVEF, left ventricular ejection fraction; LVEDD, left ventricular end-diastolic diameter; BMI, body mass index.

Among inflammatory and coagulation markers, FAR showed strong positive correlations with fibrinogen (r = 0.875) and D-dimer (r = 0.389), and strong negative correlations with albumin (r = −0.639) (all *P* < 0.001). FAR also correlated positively with monocyte count (r = 0.237, *P* < 0.001).

Regarding prognostic indicators, FAR was positively associated with NT-proBNP (r = 0.472) and Gensini score (r = 0.268) (both *P* < 0.001).

### Logistic regression analyses for predicting LVA in STEMI patients

For association analysis, multivariable logistic regression was performed based on LASSO-selected variables to identify independent factors associated with LVA (Table 3). Univariate analysis revealed significant associations between LVA and multiple variables, including age, gender, HbA1c, D-dimer, LVEDD, LVEF, Killip class ≥2, eGFR, NT-proBNP, monocyte count, S2B time, mechanical support use, IRA-LAD, Gensini score, and FAR. Multivariate analysis indicated LVEDD (*P* = 0.023), LVEF (*P* < 0.001), Killip class ≥2 (*P* < 0.001), NT-proBNP (*P* < 0.001), and IRA-LAD (*P* = 0.003) as independent predictors of LVA.

**Table 3:** Univariate and multivariate logistic regression analyses of risk factors for LVA formation.

Variables	Univariate logistic regression analysis			Multivariate logistic regression analysis		
	OR	95%CI	*P*-value	OR	95%CI	*P*-value
Age	1.02	1.01∼1.04	0.002			
Gender	0.56	0.37∼0.85	0.007			
BMI	0.96	0.90∼1.01	0.137			
Hypertension	0.8	0.56∼1.14	0.225			
Diabetes	1.39	0.93∼2.08	0.103			
HlAc(%)	1.19	1.07∼1.32	<0.001			
D-dimer	1.32	1.20∼1.46	<0.001			
LVEDD	1.23	1.19∼1.28	<0.001	1.06	1.01∼1.12	0.023
LVEF	0.73	0.69∼0.76	<0.001	0.78	0.74∼0.82	<0.001
Killip≥2	12.01	8.02∼17.98	<0.001	2.84	1.70∼4.74	<0.001
eGFR	0.98	0.97∼0.99	<0.001			
NT-proBNP	1.01	1.01∼1.01	<0.001	1.01	1.01∼1.01	0.032
Monocyte	4.53	2.86∼7.15	<0.001			
S2B	1.04	1.02∼1.05	<0.001			
No mechanical support use	0.16	0.11∼0.23	<0.001			
Non-IRA-LAD	0.12	0.07∼0.20	<0.001	0.33	0.16∼0.68	0.003
Gensiniscore	1.03	1.03∼1.04	<0.001			
FAR_T						
1		1.00 (Reference)			1.00 (Reference)	
2	2.22	1.28∼3.86	0.004	1.83	0.92∼3.65	0.087
3	4.43	2.65∼7.40	<0.001	2.1	1.09∼4.04	0.027

Variables included in the multivariable model were selected via LASSO logistic regression with 10-fold cross-validation (1-SE criterion).

LVA, left ventricular aneurysm; STEMI, ST-segment elevation myocardial infarction; OR, odds ratio; CI, confidence interval; FAR, fibrinogen-to-albumin ratio; FAR_T1, FAR tertile 1 (lowest); FAR_T3, FAR tertile 3 (highest); LVEF, left ventricular ejection fraction; NT-proBNP, N-terminal pro-B-type natriuretic peptide; LVEDD, left ventricular end-diastolic diameter; IRA-LAD, infarct-related artery as the left anterior descending artery.

LASSO initially identified 9 candidate variables, all of which were entered into multivariable logistic regression. Among these, six variables (LVEDD, LVEF, Killip class ≥2, NT-proBNP, IRA-LAD, and FAR) demonstrated independent associations with LVA (*P* < 0.05) and were retained for nomogram construction to maximize predictive accuracy. For the purpose of comparative discrimination analysis against single biomarkers, we additionally constructed a parsimonious three-variable core model comprising FAR, LVEF, and NT-proBNP. Additionally, LVEDD, Killip class ≥2, and IRA-LAD also remained significant in multivariable analysis (Table 3). After adjustment for confounders, the highest FAR tertile remained an independent predictor of LVA, with a 2.10-fold increased risk compared with the lowest tertile (OR = 2.10, 95% CI: 1.09–4.04, *P* = 0.027). The consistency between the LASSO-selected three-variable model and the full multivariable model supported the reliability of our findings, with comparable discriminative performance (AUC = 0.931 vs. 0.933).

Collinearity diagnostics showed that all variables included in the multivariable model had VIF values below 5, ranging from 1.15 for FAR to 1.92 for LVEF, indicating no significant multicollinearity among FAR and conventional infarct-severity indicators, including LVEF, NT-proBNP, and Gensini score (Supplementary Table 2).

### Subgroup analysis

**Figure 6:**
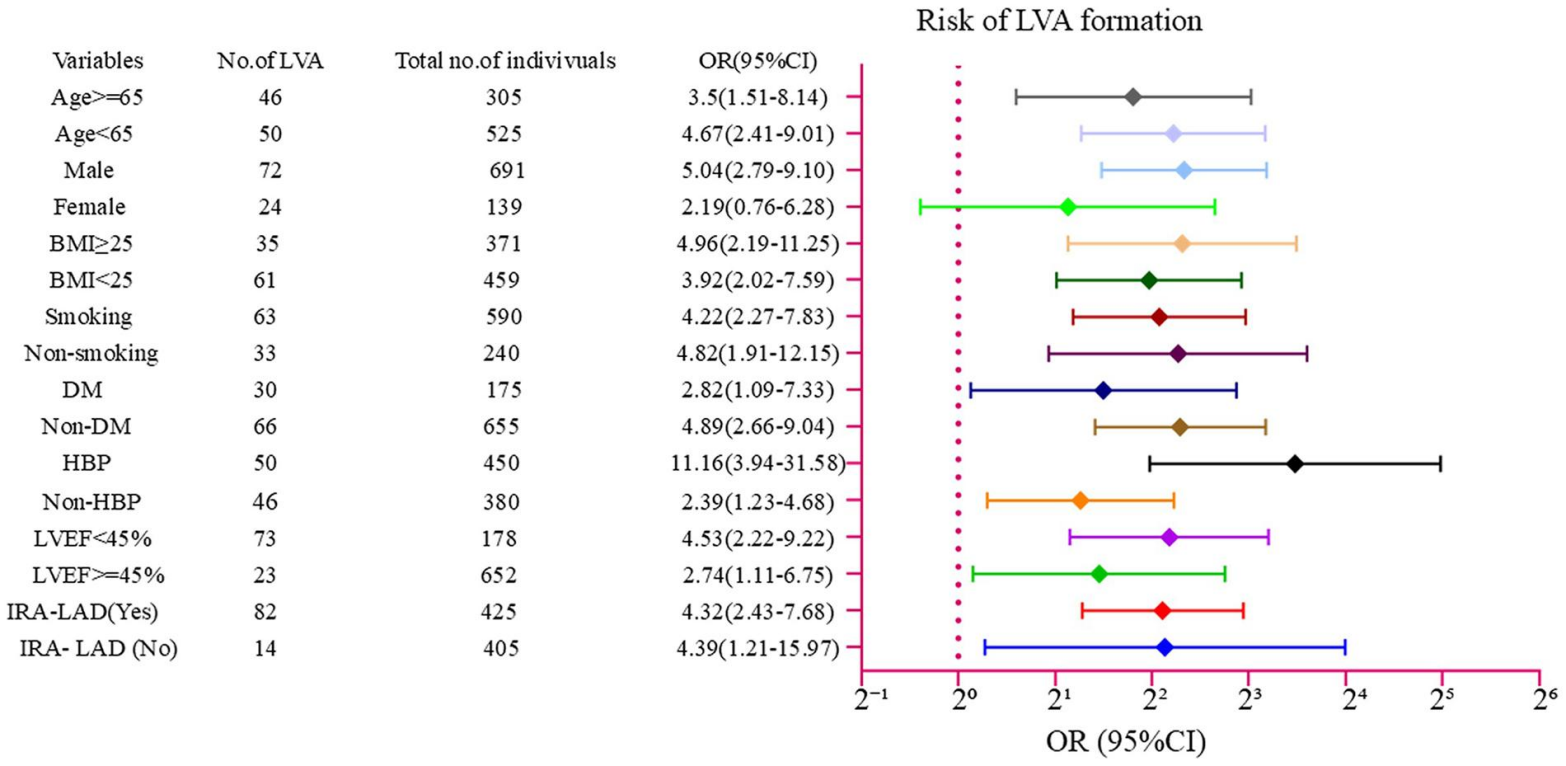
Subgroup analysis of the association between FAR index and LVA formation in patients with STEMI. FAR, fibrinogen-to-albumin ratio; LVA, left ventricular aneurysm; STEMI, ST-segment elevation myocardial infarction; OR, odds ratio; CI, confidence interval; BMI, body mass index; DM, diabetes mellitus; HBP, hypertension; LVEF, left ventricular ejection fraction; IRA-LAD, infarct-related artery as the left anterior descending artery.

Subgroup analyses were conducted to assess the independent association between FAR and LVA formation across different clinical subgroups (Figure 6). The strongest association was identified in patients with HBP (OR = 11.16, 95% CI: 3.94–31.58), followed by those with BMI ≥ 25 kg/m^2^ (OR = 4.96, 95% CI: 2.19–11.25) and non-smokers (OR = 4.82, 95% CI: 1.91–12.15). A strong positive association was also observed in patients aged < 65 years (OR = 4.67, 95% CI: 2.41–9.01) and male patients (OR = 5.04, 95% CI: 2.79–9.10).

In contrast, the association between FAR and LVA formation was relatively weaker in patients with T2DM (OR = 2.82, 95% CI: 1.09–7.33) and female patients (OR = 2.19, 95% CI: 0.76–6.28). Notably, the association in female patients did not reach statistical significance (*P* = 0.140), which is likely attributable to the smaller sample size (*n* = 139). Regarding cardiac function, the association was more pronounced in patients with reduced LVEF (LVEF <45%) (OR = 4.53, 95% CI: 2.22–9.22) compared with those with LVEF ≥45% (OR = 2.74, 95% CI: 1.11–6.75).

Finally, the association was numerically similar but with wider confidence intervals in non-LAD subgroups, likely due to the lower event rate. The effect size appeared more robust in patients with IRA-LAD (OR 4.32, 95% CI 2.43–7.68) than in those with non-LAD culprit vessels (OR 4.39, 95% CI 1.21–15.97), suggesting a potential subgroup-specific effect, with FAR exhibiting stronger predictive value in anterior myocardial infarction.

No significant interactions were observed across all subgroups, indicating that the direction and magnitude of the main findings were generally consistent regardless of age, gender, hypertension, diabetes, or other clinical characteristics. Although minor variations in effect size existed across different subgroups, the overall pattern remained stable, suggesting acceptable homogeneity and no evidence of significant heterogeneity or moderating effects in this study.

### Discriminative power analysis

**Figure 7:**
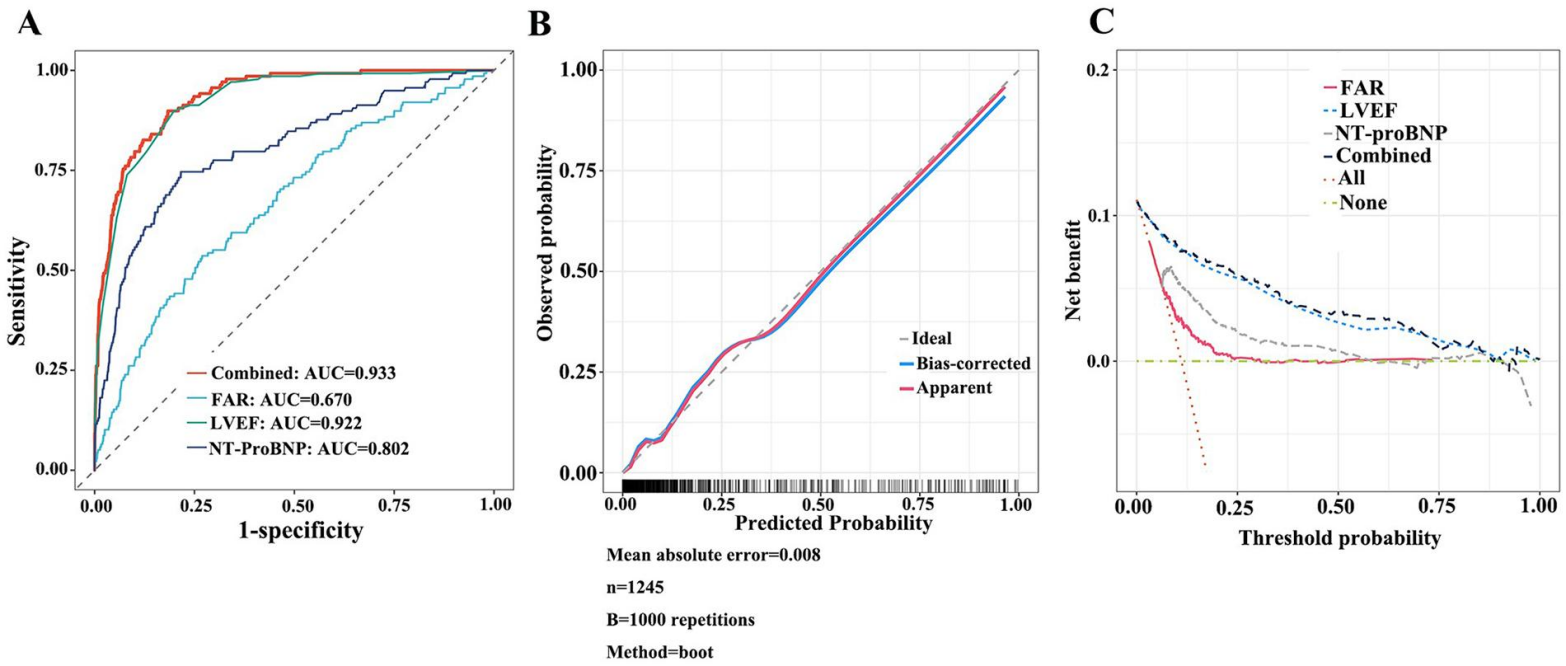
Comprehensive validation of the predictive model for LVA formation in patients with STEMI. **(A)** ROC curve analysis comparing the discriminative performance of the FAR alone, LVEF alone, NT-proBNP alone, and the combined prediction model. **(B)** Calibration curve evaluating the agreement between predicted and observed LVA probabilities. The bias-corrected curve (blue line) closely overlaps with the ideal 45° reference line (gray dashed line), with a mean absolute error (MAE) of 0.008 (*n* = 1245, 1000 bootstrap repetitions). **(C)** DCA comparing the net clinical benefit of Model 1 (FAR), Model 2 (LVEF), Model 3 (NT-proBNP), and the combined model (FAR + LVEF + NT-proBNP). The combined model shows greater net benefit across nearly all threshold probabilities, outperforming all individual models and both the “treat all” and “treat none” reference lines. AUC, area under the curve; CI, confidence interval; FAR, fibrinogen-to-albumin ratio; LVEF, left ventricular ejection fraction; NT-proBNP, N-terminal pro-B-type natriuretic peptide; LVA, left ventricular aneurysm; STEMI, ST-segment elevation myocardial infarction; DCA, decision curve analysis.

For predictive modeling, we constructed and validated a prediction model for LVA risk assessment. ROC analysis assessed discriminative performance for LVA prediction (Figure 7A, Table 4). FAR showed moderate discrimination (AUC 0.670, 95% CI 0.621–0.718), NT-proBNP demonstrated good performance (AUC 0.802, 95% CI 0.759–0.845), and LVEF exhibited high discrimination (AUC 0.922, 95% CI 0.901–0.944). The combined model achieved the highest AUC (0.933, 95% CI 0.914–0.952), significantly higher than those of each individual marker (all *P* < 0.001).

**Table 4:** Analysis of the ROC curve for predictive ability of LVA formation.

Variables	AUC	SE	Sensitivity	Specificity	95%CI
FAR	0.67	0.025	0.5362	0.7308	0.621–0.718
NT-proBNP	0.802	0.022	0.7464	0.7841	0.759–0.845
LVEF	0.922	0.011	0.8986	0.8013	0.901–0.944
Composite variable	0.933	0.01	0.8986	0.8157	0.914–0.952

AUC, area under the receiver operating characteristic curve; CI, confidence interval; FAR, fibrinogen-to-albumin ratio; LVEF, left ventricular ejection fraction; NT-proBNP, N-terminal pro-B-type natriuretic peptide.

**Table 5:** Predictive performance of the model in the temporal validation cohort variables.

Variables	AUC	SE	Sensitivity	Specificity	95% CI
FAR	0.729	0.060	0.633	0.804	0.613–0.844
NT-proBNP	0.864	0.043	0.867	0.814	0.781–0.948
LVEF	0.927	0.018	0.933	0.857	0.892–0.962
Composite variable	0.934	0.019	0.933	0.875	0.894–0.974

FAR, fibrinogen-to-albumin ratio; LVEF, left ventricular ejection fraction; NT-proBNP, N-terminal pro-B-type natriuretic peptide; LVA, left ventricular aneurysm; AUC, area under the curve; CI, confidence interval.

Calibration analysis showed excellent agreement between predicted and observed probabilities (MAE of 0.008), with bias-corrected curves closely aligned to the ideal line following 1000 bootstrap resamples (Figure 7B).

DCA was performed to compare the net clinical benefit of FAR alone, LVEF alone, NT-proBNP alone, and the combined model (Figure 7C). All models yielded positive net benefit exceeding the “treat none” reference line. The combined model exhibited greater net benefit across nearly the entire threshold probability interval (0–0.8) than all single predictors and both reference strategies, indicating good clinical utility for LVA risk stratification.

### Development of a nomogram for predicting LVA risk

**Figure 8:**
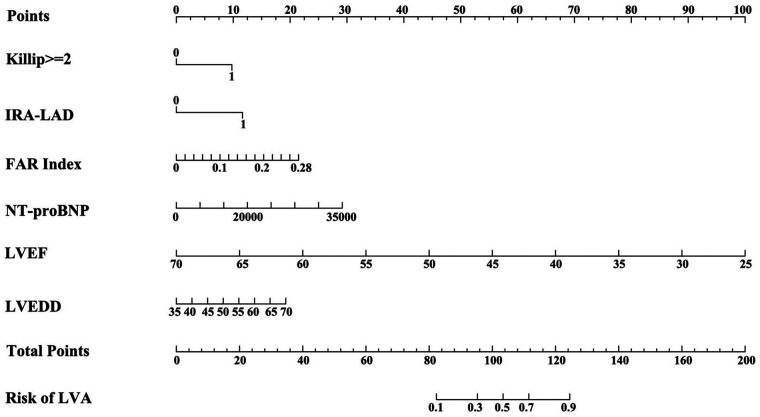
Nomogram for predicting the risk of LVA in STEMI patients undergoing pPCI. This nomogram was developed to translate the final multivariate logistic regression model into a clinically applicable tool for bedside risk stratification. LVA, left ventricular aneurysm; STEMI, ST-segment elevation myocardial infarction; FAR, fibrinogen-to-albumin ratio; NT-proBNP, N-terminal pro-B-type natriuretic peptide; LVEF, left ventricular ejection fraction; LVEDD, left ventricular end-diastolic diameter; IRA-LAD, infarct-related artery as the left anterior descending artery; CI, confidence interval.

Based on the association analysis and predictive modeling, a clinical nomogram was developed. The nomogram incorporating all six independent predictors identified in multivariable analysis—Killip grade ≥ II, IRA-LAD, FAR, NT-proBNP, LVEF, and LVEDD—was constructed to predict LVA probability in STEMI patients undergoing pPCI (Figure 8). These variables represented the full set of LASSO-screened candidates that retained statistical significance in multivariable regression, ensuring full consistency across analytic steps.

### Temporal validation of the prediction model

To further address model generalizability, we performed temporal validation. Baseline characteristics of the development and temporal validation cohorts are shown in Supplementary Table 1. The established prediction model was applied to the temporal validation cohort without refitting or reselecting predictors.

In the temporal validation cohort, FAR alone showed moderate discrimination for LVA prediction, with an AUC of 0.729 (95% CI: 0.613–0.844). LVEF and NT-proBNP demonstrated higher discriminative ability, with AUCs of 0.927 (95% CI: 0.892–0.962) and 0.864 (95% CI: 0.781–0.948), respectively. The combined model retained excellent discriminative performance, with an AUC of 0.934 (95% CI: 0.894–0.974) (Table 5 and Supplementary Figure S1A). Calibration analysis showed acceptable agreement between predicted and observed LVA probabilities, with a mean absolute error of 0.021 after 1,000 bootstrap resamples (Supplementary Figure S1B). DCA further indicated that the combined model provided greater net benefit than individual predictors across a broad range of clinically relevant threshold probabilities (Supplementary Figure S1C).

In addition, RCS analysis in the temporal validation cohort showed a generally positive association between FAR and LVA risk, although the confidence interval widened at higher FAR values because of the limited number of events (Supplementary Figure S2).

## Discussion

This study was designed to develop and temporally validate a clinical prediction model for LVA in patients with STEMI undergoing pPCI. The main findings were as follows. First, FAR was independently associated with LVA after adjustment for clinically relevant variables. Second, a clinical nomogram incorporating Killip grade ≥ II, IRA-LAD, FAR, NT-proBNP, LVEF, and LVEDD showed good discrimination, calibration, and clinical net benefit for individualized LVA risk assessment. Third, temporal validation in a later cohort showed preserved model performance, supporting the temporal stability of the prediction model within the same clinical setting.

Temporal validation was performed to address potential optimism associated with internal validation alone. In the temporal validation cohort, the combined model retained excellent discrimination and acceptable calibration, and decision curve analysis suggested potential clinical net benefit across clinically relevant threshold probabilities. These findings partly address concerns regarding overoptimism from model development and validation within the same dataset. Nevertheless, because both the development and temporal validation cohorts were derived from the same institution, further multicenter external validation remains necessary before broad clinical application.

Inflammation and coagulation activation play important roles in adverse ventricular remodeling and LVA development after myocardial infarction (26–28). Fibrinogen is an acute-phase reactant involved in inflammation and thrombosis (13), whereas albumin has anti-inflammatory and antioxidant properties (29). FAR therefore reflects the balance between pro-inflammatory/coagulative activity and anti-inflammatory status. Previous studies have demonstrated the prognostic value of FAR in various cardiovascular diseases, including heart failure (20) and acute coronary syndrome (30). Our findings extend these observations by suggesting that FAR may serve as a readily available inflammatory/coagulative marker for LVA risk stratification in patients with STEMI.

Patients with higher FAR also had higher NT-proBNP levels, lower LVEF, and higher Gensini scores, suggesting that FAR may partly reflect greater infarct severity and the systemic response to myocardial injury. However, FAR remained independently associated with LVA after adjustment for ventricular function, myocardial stress, coronary lesion severity, and other clinically relevant variables. In addition, collinearity diagnostics showed that all VIF values were below 5, indicating no significant multicollinearity among FAR, LVEF, NT-proBNP, and Gensini score. The association between FAR and LVA was also directionally consistent across LVEF subgroups and was further supported by temporal validation, in which FAR alone retained moderate discriminative ability. These findings suggest that FAR is not merely redundant with conventional infarct-severity indicators, but may provide complementary information related to inflammatory and coagulative status. Nevertheless, FAR may still partly reflect the overall severity of STEMI, and residual confounding cannot be fully excluded in this retrospective observational study.

Patients in the development cohort received contemporary guideline-directed medical therapy, including statins, angiotensin-converting enzyme inhibitors/angiotensin receptor–neprilysin inhibitors, beta-blockers, and mineralocorticoid receptor antagonists. These therapies were considered as potential confounding variables because of their established influence on post-infarction ventricular remodeling. After adjustment for clinical covariates in multivariable analysis, FAR remained independently associated with LVA, suggesting that the association between FAR and LVA is less likely to be solely explained by differences in standard cardioprotective pharmacotherapy.

LVEF is a well-established predictor of adverse outcomes in STEMI patients (31), and our study confirmed its strong discriminative ability for LVA prediction. NT-proBNP, a marker of myocardial stress and ventricular dysfunction (32), also demonstrated good predictive performance. After incorporating FAR and NT-proBNP, the combined model achieved a slightly higher AUC than LVEF alone. However, this numerical increase was modest, and the clinical value of the combined model should not be interpreted solely on the basis of AUC improvement. Rather, the rationale for the combined model lies in the integration of complementary pathophysiological information. LVEF reflects ventricular systolic function, NT-proBNP reflects myocardial wall stress and ventricular dysfunction, whereas FAR reflects systemic inflammatory and coagulative status. These dimensions are biologically related but not identical in the development of post-infarction ventricular remodeling and LVA formation. In addition to discrimination, calibration analysis, decision curve analysis, and temporal validation further supported the potential usefulness of the combined model for individualized LVA risk assessment.

Subgroup analyses showed generally consistent directions of association across clinically relevant strata, including LVEF subgroups. However, these analyses were exploratory and hypothesis-generating. Because multiple subgroup comparisons were performed without formal multiplicity adjustment, false-positive subgroup findings cannot be excluded. Therefore, subgroup results should be interpreted cautiously and should not be regarded as confirmatory evidence of subgroup-specific effects.

Several previous studies have investigated inflammatory markers for predicting LVA and other adverse cardiovascular events. For example, C-reactive protein (33) and fibrosis-4 (34) have been associated with increased LVA risk and high thrombus burden (35). In addition, Lokman Hekim et al. reported that the C-reactive protein-serum albumin ratio was an independent risk factor for thrombus burden (36). However, the clinical application of some inflammatory markers may be limited by variable cut-off values and lack of standardization. In contrast, FAR can be calculated from routine laboratory tests without additional specialized assays. This makes FAR a simple and cost-effective marker that may assist preliminary risk stratification in STEMI care. Future prospective multicenter studies are warranted to validate the predictive value of FAR and to determine whether FAR-guided risk assessment can improve clinical decision-making and patient outcomes.

### Limitations

Our study has several limitations. First, although temporal validation was performed in a chronologically independent cohort of patients admitted after the model-development period, both the development and validation cohorts were derived from the same center. Therefore, the generalizability of the model to other institutions, regions, and healthcare settings remains to be further confirmed in larger multicenter external validation studies. Second, the number of LVA events in the temporal validation cohort was relatively limited, which may affect the stability of calibration and decision curve estimates. Third, some potential confounders, such as medication adherence and lifestyle factors, were not fully collected or adjusted for in the analysis. Fourth, we only assessed short-term outcomes, and longer follow-up is needed to confirm the prognostic value of FAR. Finally, the exact timing of first diagnosis of LVA was not systematically recorded for each patient, which prevented us from conducting further survival analyses to explore the dynamic relationship between FAR and LVA risk.

### Future directions

Despite these limitations, our study indicates that FAR may serve as a useful predictor for LVA in STEMI patients. Further multicenter prospective studies are needed to validate these findings and determine optimal cutoff values of FAR. The underlying mechanisms linking FAR to LVA also warrant further investigation. Our constructed nomogram may represent a promising clinical tool, but its clinical value and effect on patient outcomes require further validation in practice.

## Conclusion

In conclusion, this study demonstrates that FAR is an independent, readily accessible, and clinically meaningful predictor of LVA formation in STEMI patients. The integration of FAR into a comprehensive nomogram showed high discriminative power, favorable calibration, and positive net clinical benefit. Additional temporal validation in a later cohort further supported the stability of the model, although multicenter external validation remains warranted before broad clinical implementation.

## Data Availability

The raw data supporting the conclusions of this article will be made available by the authors, without undue reservation.

## Supplementary material

Supplementary Figure S1 Temporal validation of the prediction model for left ventricular aneurysm formation in patients with STEMI (A) ROC curves comparing the discriminative performance of FAR alone, LVEF alone, NT-proBNP alone, and the combined prediction model in the temporal validation cohort. (B) Calibration curve evaluating the agreement between predicted and observed probabilities of LVA in the temporal validation cohort. The bias-corrected curve was generally close to the apparent curve and the ideal reference line, indicating acceptable calibration of the prediction model, with a MAE of 0.021 after 1,000 bootstrap resamples (*n* = 310). (C) DCA comparing the clinical net benefit of FAR alone, LVEF alone, NT-proBNP alone, and the combined prediction model across a range of threshold probabilities. STEMI, ST-segment elevation myocardial infarction; LVA, left ventricular aneurysm; FAR, fibrinogen-to-albumin ratio; LVEF, left ventricular ejection fraction; NT-proBNP, N-terminal pro-B-type natriuretic peptide; ROC, receiver operating characteristic; AUC, area under the curve; DCA, decision curve analysis; MAE, mean absolute error.

Supplementary Figure S2 RCS analysis of the association between FAR index and LVA risk in the temporal validation cohort. FAR, fibrinogen-to-albumin ratio; LVA, left ventricular aneurysm; OR, odds ratio; CI, confidence interval.
