# Supplementary figures and images for: Fibrinogen-to-albumin ratio independently predicts left ventricular aneurysm in STEMI patients: a retrospective cohort study
Source: Front Cardiovasc Med. 2026 Jul 8;13:1837371. doi: 10.3389/fcvm.2026.1837371 (PMC13388391; doi:10.3389/fcvm.2026.1837371)

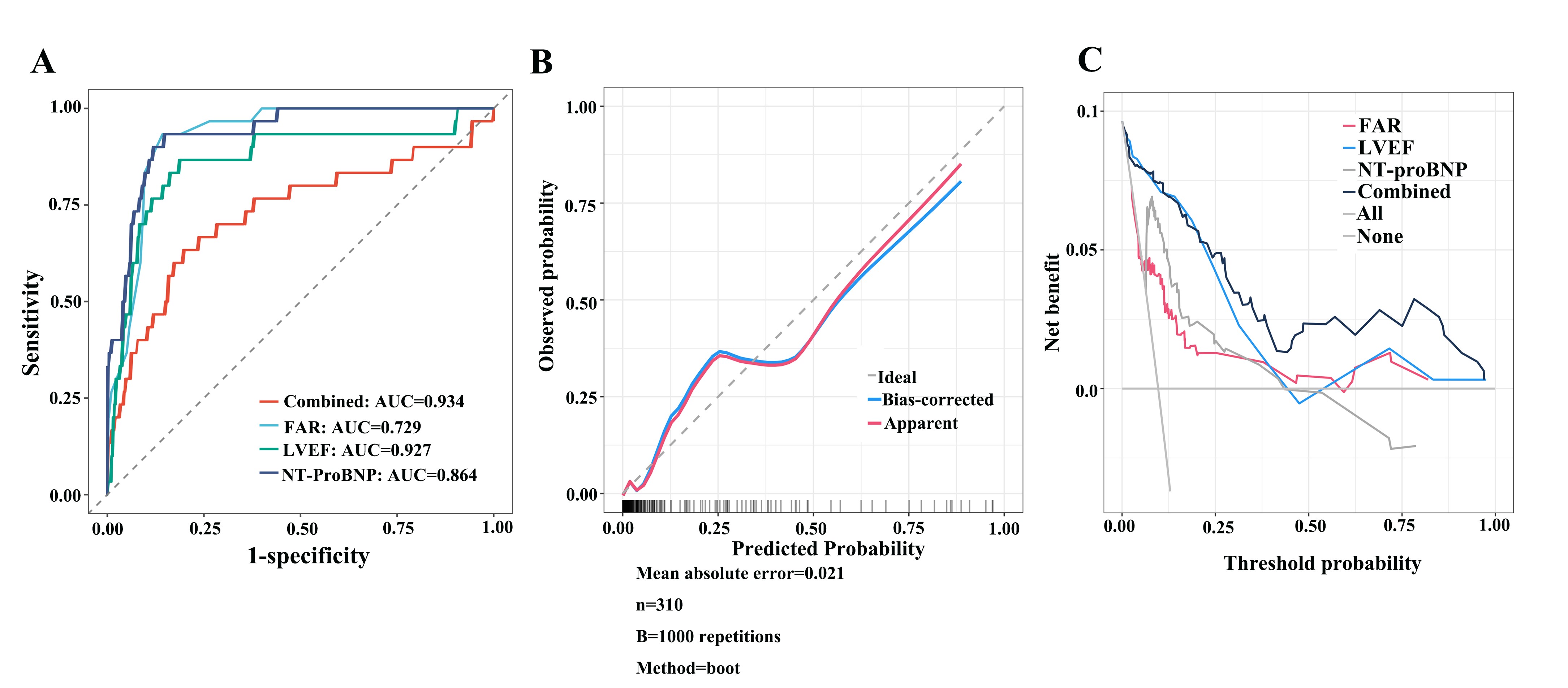

Supplement: Supplementary Figure S1 — Temporal validation of the prediction model for left ventricular aneurysm formation in patients with STEMI (A) ROC curves comparing the discriminative performance of FAR alone, LVEF alone, NT-proBNP alone, and the combined prediction model in the temporal validation cohort. (B) Calibration curve evaluating the agreement between predicted and observed probabilities of LVA in the temporal validation cohort. The bias-corrected curve was generally close to the apparent curve and the ideal reference line, indicating acceptable calibration of the prediction model, with a MAE of 0.021 after 1,000 bootstrap resamples (n = 310). (C) DCA comparing the clinical net benefit of FAR alone, LVEF alone, NT-proBNP alone, and the combined prediction model across a range of threshold probabilities. STEMI, ST-segment elevation myocardial infarction; LVA, left ventricular aneurysm; FAR, fibrinogen-to-albumin ratio; LVEF, left ventricular ejection fraction; NT-proBNP, N-terminal pro-B-type natriuretic peptide; ROC, receiver operating characteristic; AUC, area under the curve; DCA, decision curve analysis; MAE, mean absolute error. [file Image1.jpeg]

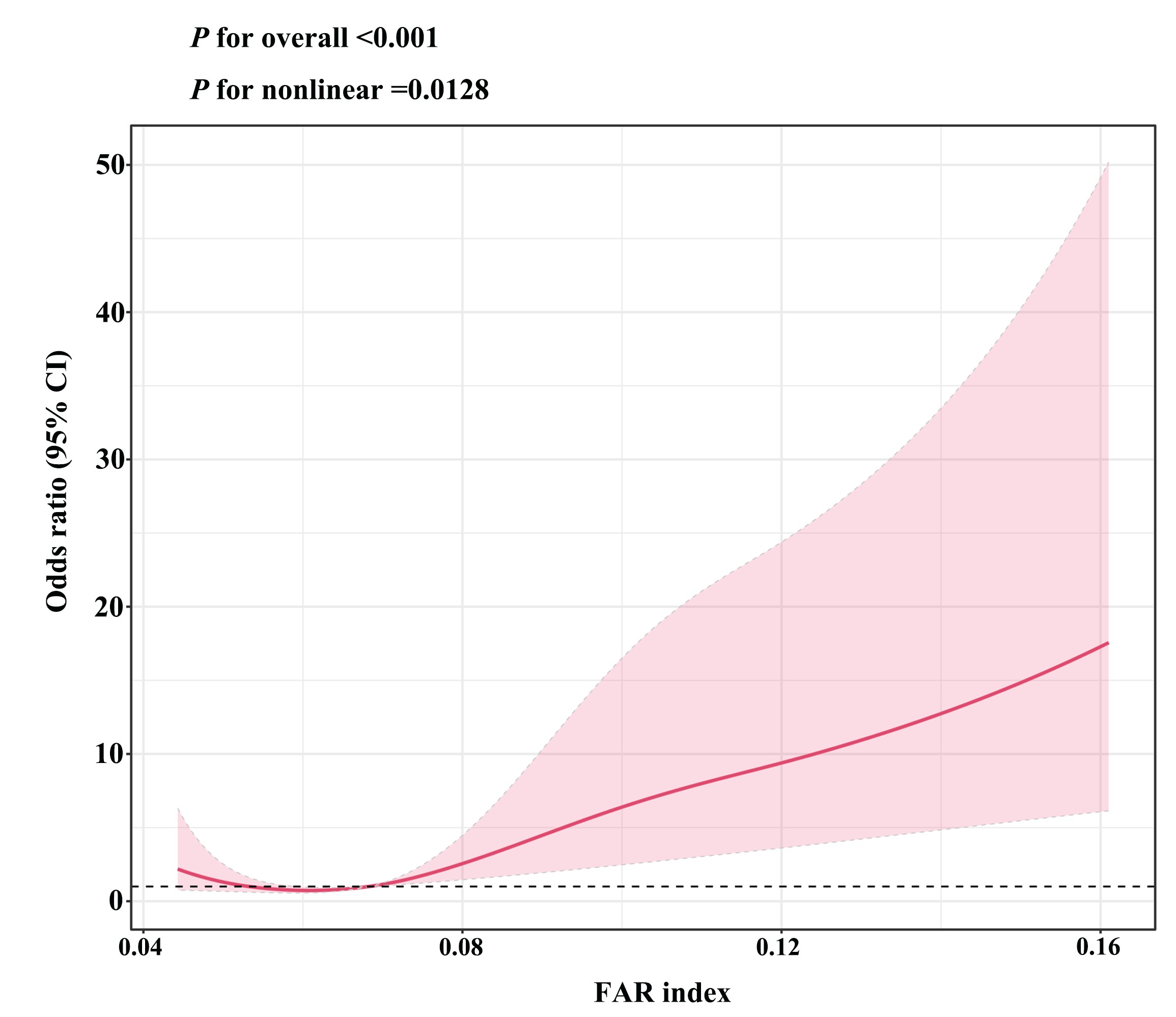

Supplement: Supplementary Figure S2 — RCS analysis of the association between FAR index and LVA risk in the temporal validation cohort. FAR, fibrinogen-to-albumin ratio; LVA, left ventricular aneurysm; OR, odds ratio; CI, confidence interval. [file Image2.jpeg]
